# Auditory Brainstem Responses to Successive Sounds: Effects of Gap Duration and Depth

**DOI:** 10.3390/audiolres11010005

**Published:** 2021-01-28

**Authors:** Fan-Yin Cheng, Craig A. Champlin

**Affiliations:** Department of Speech, Language and Hearing Sciences, University of Texas at Austin, Austin, TX 78712, USA; champlin@austin.utexas.edu

**Keywords:** temporal processing, gap duration, gap depth, successive sounds, auditory evoked potentials

## Abstract

Temporal acuity is the ability to differentiate between sounds based on fluctuations in the waveform envelope. The proximity of successive sounds and background noise diminishes the ability to track rapid changes between consecutive sounds. We determined whether a physiological correlate of temporal acuity is also affected by these factors. We recorded the auditory brainstem response (ABR) from human listeners using a harmonic complex (S1) followed by a brief tone burst (S2) with the latter serving as the evoking signal. The duration and depth of the silent gap between S1 and S2 were manipulated, and the peak latency and amplitude of wave V were measured. The latency of the responses decreased significantly as the duration or depth of the gap increased. The amplitude of the responses was not affected by the duration or depth of the gap. These findings suggest that changing the physical parameters of the gap affects the auditory system’s ability to encode successive sounds.

## 1. Introduction

Identifying a sequence of acoustic events is an essential part of perceiving a sound stream [1]. The sound wave’s temporal envelope provides a potentially useful physical cue that may facilitate the segmentation of a sound sequence such as neighboring speech sounds (phonemes), musical notes, or environmental warning signals. Fluctuation in the envelope creates gaps or partial gaps, and these likely provide valuable markers to distinguish successive sounds. By detecting the differences between relevant segments, the brain decodes the incoming acoustic characteristics and parses the stream into meaningful units. Moreover, temporal acuity is the ability of human listeners to differentiate the successive segments.

**Figure 1:**
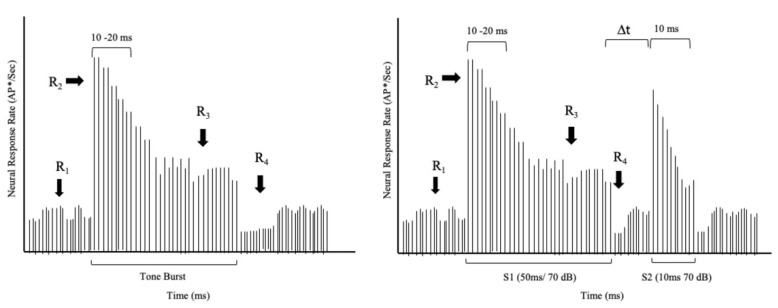
Single auditory fiber response to a single tone (**left** panel). Individual fiber response to successive tones (**right** panel). Single fiber response to successive tones (* AP denotes action potentials). (Adapted from Pickles, 2013, pp. 76–90.).

How does the auditory nervous system process sound segments, or, more specifically, how might single neurons respond to successive sounds? Pickles [2] described how individual auditory neurons respond to a tonal stimulus. As shown schematically in Figure 1, a tone burst presented above the neuron’s threshold produces an abrupt increase in response rate (R2, increasing rate) at the tone’s onset. A rapid decline follows the increasing rate until a stable steady-state rate (R3, steady rate) is achieved. The steady state persists throughout the tone. At the tone’s offset, there is another abrupt decline (R4) in response rate, and the neuron quickly returns to its spontaneous rate (R1, spontaneous rate). Figure 1 (left panel) reveals the sharp onset response at the beginning of a tone burst, the response decline, and a transient offset at the end of the stimulus.

With this differential rate mechanism, a neuron can respond to two tones presented in close succession. A given neuron responds to the first tone in a manner described in Figure 1. If a second tone with comparable frequency content is delivered immediately following the first tone’s offset, so there is no gap of silence, the neuron will not have sufficient time to recover. Consequently, it will be unable to muster an onset response (see Figure 1). If the second tone is identical to the first one, the neuron will resume responding at the steady-state rate. However, if an adequate gap of silence is interposed between the two tones, the neuron will recover its capability to produce an onset response to the second tone.

Further, the magnitude of this onset response will depend on the length of the gap—the longer the gap, the higher the increase in rate. Zhang et al. [3] demonstrated this phe-nomenon by recording activity from single neurons. They measured a single neuron’s response to finding the minimal gap between tone burst and noise burst for encoding these successive sounds into two acoustic segments. By changing the intensity of stimuli and the duration of the gap, they found the temporal gap threshold is 2–3 ms in the chinchilla’s neural responses across different stimulus intensities. A comparable gap threshold has been obtained psychoacoustically in human listeners. For example, Smiarowski and Carhart [4] measured temporal acuity by playing sequential pairs of noise bursts while adjusting the gap between noise bursts within each pair. These investigators found that the minimum gap that can be perceived by typical listeners is approximately 2.5 ms, which is consistent with the physiological findings from single neurons.

The auditory brainstem response (ABR) technique is used widely to record the neural activity generated in subcortical pathways [5,6,7]. Previous studies have been performed with successive sounds, such as click or tone-bursts [8,9]. These studies indicated that neurons respond differentially at the subcortical level. Further, acoustic events often are not identical sound combinations. For example, speech syllables are composed of transient and sustained components that could be differentiated with the different phonemic characteristics between them and the amplitude fluctuation from one to the other.

**Figure 2:**
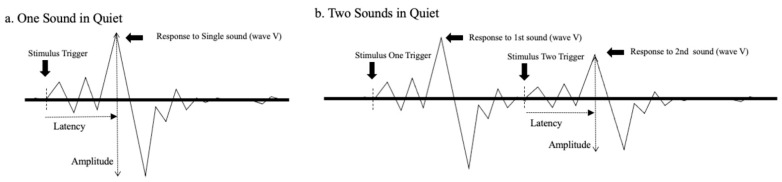
Simple diagrams of ABR waveforms evoked by a brief tone burst (panel **a**) or successive tone bursts (panel **b**). Major peaks of the responses are present and wave V is labeled. The measures of latency and amplitude of wave V are shown in each stimulus configuration. Latency is measured from the stimulus trigger to the peak’s maximum.

Figure 2 was created to illustrate how neural responses, as revealed by the ABR, could be affected by a successive sound. Panel “a” of the figure shows a schematized ABR waveform resulting from transient stimulation. The peaks and valleys that characterize the response are visible including wave V, which is generally the most robust peak. The typical measurements of response latency (from stimulus onset to the local maximum) and amplitude (from the local maximum to the following local minimum) are also indicated. Panel “b” shows ABRs to two consecutive stimuli. The waveform on the left side is the response to the initial stimulus, while the waveform on the right side is the response to the subsequent stimulus. Notice that the response to the second sound is somewhat diminished (i.e., decreased amplitude and increased latency) presumably due to alterations in neural responsiveness mentioned previously. Further, we hypothesize that the second ABR elicited would be influenced by a brief pause or gap interposed between the first and second sounds.

Of interest in the present study was whether a physiological response could be measured noninvasively in humans to reveal gap sensitivity. In other words, could a physiological correlate of temporal acuity be identified? Previous studies by Burkard and Deegan [8] and Marler and Champlin [9] reported that a leading sound influenced the ABR evoked by a trailing sound. For example, Marler and Champlin [9] showed the latency of wave V increased significantly when the signal followed a masking noise compared to when the preceding noise was absent. These investigators, however, did not explore the effects of the specific parameters of the gap between the two sounds. Therefore, our goal was to examine the effects of a preceding sound (S1) on a successive sound (S2) in different temporal contexts using the ABR. As the duration and depth of the gap were manipulated, we investigated (1) how the length of a gap between sounds influences auditory processing in brainstem responses, and (2) how the intensity of background noise influences sensory processing in brainstem responses. Specifically, using successive sounds to mark a temporal gap, our objective was to determine whether the latency and/or amplitude of the ABR were affected by the length and depth of the gap. If one or both of these manipulations altered measures of the ABR, then we may have identified an objective measure of gap detection that potentially could be used in clinical situations where assessing temporal resolution is not possible via behavioral methods. Moreover, this study’s results could provide a picture of how neurons in the brainstem respond to different sound pairs that have similar acoustical traits.

## 2. Method

### 2.1. Participants

The study included twelve adults (six men and six women) between the ages of 18–35 years. All participants had typical auditory function revealed by otoscopy, middle-ear analysis, and a hearing screening. Participants were excluded if they have a history of hearing, speech, or language disorders. Participants were required to present thresholds ≤25 dB HL at each octave within the clinical frequency range 150–8000 Hz to be defined as normal hearing for study inclusion. All participants provided written informed consent to participate in the study. The University’s Institutional Review Board approved all procedures.

### 2.2. Stimuli

Stimuli were composed of a sound (S1) followed by the target signal (S2). S1 was 50 ms in duration and gated with 10 ms cosine-squared rise-fall windows. S1 was a harmonic complex with ten harmonics ranging from 200 to 2 kHz in 200-Hz frequency increments. The target signal (S2) was a 10-ms 1 kHz tone, which is the fifth harmonic of the S1, shaped with a 5-ms cosine-squared rise-fall window. A silent gap of variable length was interposed between S1 and S2. The overall stimulus rate was fixed at 7 Hz, and thus the duration of a stimulus cycle was about 143 ms. The presentation level of S1 and S2 was 70 decibels sound pressure level (dB SPL), which approximated the level of normal conversational speech. When used, the background noise followed a Gaussian distribution and had a bandwidth of 20 kHz. The noise was presented continuously. Stimuli were delivered to the right ear via an electrically shielded insert earphone (Etymotic Research, model ER-3A). Signals and noises were calibrated using dB sound pressure level (dB SPL) with a sound level meter (Quest, model 1800) connected to a 2-cc coupler (GRAS, model RA0113) that included a 1-inch pressure microphone (GRAS, model 40EN).

**Figure 3:**
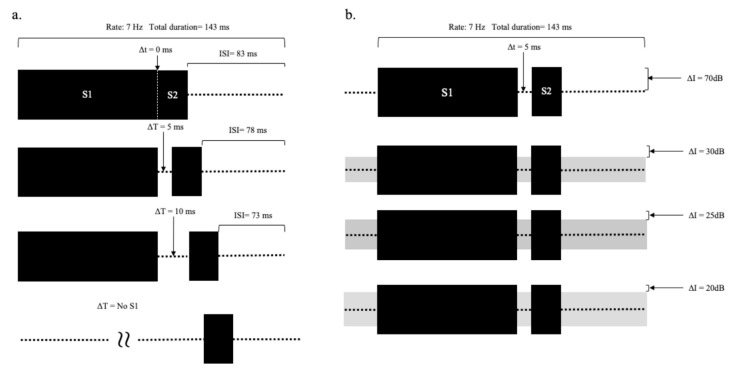
Diagrams of two tests. (**a**) Different durations of the silent gap (∆t). (**b**) Different intensities of continuous background noise (gray blocks) (∆I) (interstimulus interval (ISI) = 143 ms − (S1 + S2 + ∆t)).

Two experiments were conducted. In Experiment 1, ABRs were acquired at different durations of the silent gap (∆t). The stimuli (see Figure 3a) included silent gaps (∆t) ranging from 0 to 10 ms in steps of 5 ms (0, 5, 10 ms in ∆t), and a condition where S1 was not present, and thus the gap was considered infinite.

In Experiment 2, ABRs were obtained at different intensities of continuous background noise. The background noise partially filled the gap between S1 and S2, so varying the noise level changed the gap depth. The silent gap was fixed at 5 ms. The intensities of S1 and S2 were fixed at 70 dB SPL (see Figure 3b), and a continuous noise was presented at 0, 40, 45, and 50 dB SPL. The gap depth was defined as the difference between the target signal level and continuous noise levels; therefore, when S2 was 70 dB SPL, the gap depths (∆I) were 70, 30, 25, and 20 dB, respectively. The tones and noise were added together and delivered to the right ear. A control condition was also run in the absence of any perceptible sound.

### 2.3. Recording

The ABRs were obtained with a single-channel recording system (Intelligent Hearing Systems, model Smart EP system). Three gold-plated electrodes were used, and they were applied to the forehead and each earlobe. The electrodes were held in place with a small (2 cm^2^) piece of surgical tape. The recordings were amplified (gain = 100 k), filtered (0.1–2 kHz), and then digitized at a sampling rate of 40 kHz. A total of 1024 presentations of each stimulus were averaged over a 102.4-ms epoch for each stimulus condition, including the duration of 50 ms S1, 10 ms S2, gap duration, and the pre-stimulus interval. As the presentation rate should not exceed a rate of 7.55 stimuli per second, this study used the rate of 7 stimuli per second. Therefore, each run took around two mins (1024/7 = 146 s). Thus, the total time for data collection was approximately 80 min (one syllable × 16 sets (seven sets for ∆t and nine sets for ∆I) × two runs/set × 2.5 min/run). Each participant was tested in a single experimental session, which lasted for about 100-min (read and sign consent form = 5-min; hearing screening = 5-min; electrode application = 8-min; data collection = 80-min).

During the recording phase, the participant was instructed to rest quietly but remained awake in a reclining chair located in a sound-treated booth. Brief rest breaks were provided at 15-min intervals to help maintain wakefulness. Following the session, the electrodes were gently removed, the electrode gel was wiped off, and the participant was excused.

### 2.4. Data Analysis

The study involved two independent variables: gap duration and gap depth. The dependent variables were the amplitude and latency of wave V of the ABR. The ABR wave V peaks were selected based on those observed during pilot testing and by comparing the acquired wave to the control ABR waveform. Wave V amplitude was defined as the difference between the amplitude at the response peak (i.e., local maximum) and the valley immediately following the peak (i.e., local minimum). Wave V latency was measured at the peak of the response. Two criteria were used to determine the presence of ABR wave V: (a) if the maximal positive amplitude occurred within a 7–13 ms window after the onset of S2, and (b) if the absolute amplitude of wave V was greater than two times the amplitude of the average pre-stimulus activity. The latencies were calculated relative to the onset of S2.

**Figure 4:**
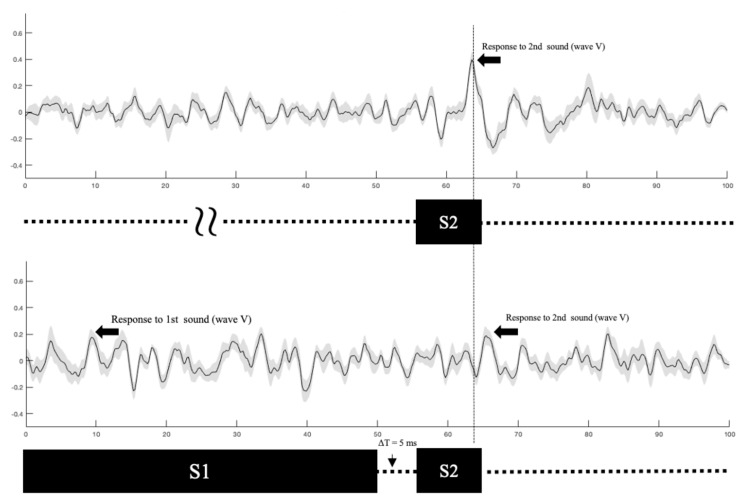
Grand mean (*n* = 12) ABR waveforms for the S2-alone condition (**upper** panel) and S1-followed-by-S2 condition (**lower** panel). Gray shading of each waveform represents ± one standard deviation from the mean. The stimulus configuration appears below each waveform. Wave V is indicated with arrows. The vertical dashed line spanning both waveforms reveals the effect of S1 on response latency.

Our measurement process is illustrated in Figure 4 where ABR waveforms are plotted for two stimulus conditions in Experiment 1 where gap duration varied. The stimulus configuration is shown immediately below each waveform. The longer-duration first stimulus (S1) is followed by the shorter second stimulus (S2). The gap between S1 and S2 is also shown (∆t = 5 ms). The upper panel shows the grand mean ABR evoked by S2 alone. Wave V is identified; latency measurements were made relative to the onset of S2. The lower panel shows the grand mean ABR elicited by S2 when S1 preceded it. Again, wave V is marked. A vertical line is provided as a reference and reveals the response latency shift in the S1 + S2 condition (lower panel) relative to the S1-alone condition (upper panel). The results reported below were obtained in a similar manner, namely the amplitude and latency of wave V in response to S2 were measured under various conditions of gap duration and depth.

## 3. Results

This study examined temporal encoding by varying the duration and depth of a silent gap between a preceding sound and target signal. By investigating the effects of the gap on target signal at the subcortical level, we gained a basic understanding of how listeners with normal hearing respond to sounds presented successively.

### 3.1. Experiment 1: Gap Duration (∆t)

**Figure 5:**
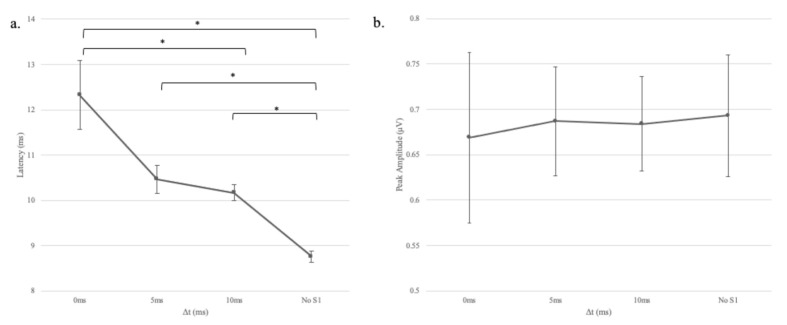
Mean response latencies (panel **a**) and amplitudes (panel **b**) for different gap durations (∆t). Error bars indicate +/– one standard deviation from the mean. One asterisk represents *p* < 0.05.

The mean response latencies and amplitudes for different gap durations are shown in panels “a” and “b,” respectively of Figure 5. As revealed in the figure, response latency increased as the gap duration decreased. To quantify the duration effect, a one-factor analysis of variance with repeated measures was conducted to assess the impact of gap duration (∆t = 0, 5, 10, and 50 ms) on participants’ response latency and amplitude values. A significant effect of ∆t on response latency, Wilks’ Lamda = 0.08, F(3, 8) = 31.63, *p* < 0.001, η^2^ = 0.92, suggesting that gap duration affected the response to the successive stimulus (S2). The effect size, calculated using eta squared, indicated that gap duration accounted for 92% of the variance in response latency. To follow-up on the significant duration effect, LSD post hoc tests were performed. The post-hoc tests showed that response latencies were significantly shorter in the no-S1 condition compared to 0, 5, and 10-ms gap conditions. The latency for the 10-ms condition was also significantly shorter than the 0-ms condition. Somewhat unexpectedly, there was no significant effect of ∆t on response amplitude, Greenhouse-Geisser = 0.63, F(2.74, 27.36) = 2.14, *p* = 0.12, η^2^ = 0.18, suggesting that gap duration did not affect the response to the successive stimulus (S2). Moreover, no clear trend was observed in response amplitude with changes in gap duration.

### 3.2. Experiment 2: Gap Depth (∆I)

**Figure 6:**
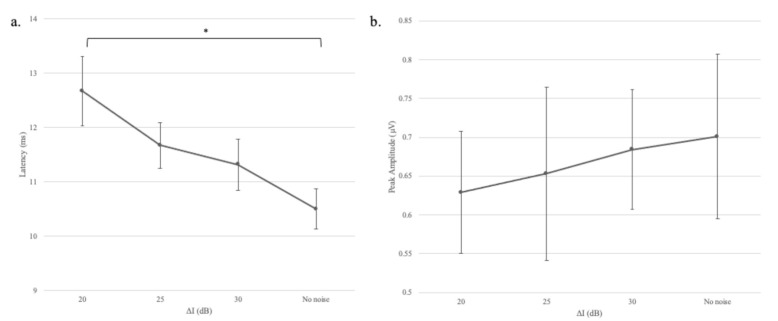
Mean response latencies (panel **a**) and amplitudes (panel **b**) for different gap depths (∆I). Error bars indicate +/– one standard deviation from the mean. One asterisk represents *p* < 0.05. The no-noise condition refers to ∆I = 70 dB and the continuous noise was absent.

The mean response latencies and amplitudes for different gap depths are plotted in panels “a” and “b,” respectively in Figure 6. The figure shows that response latency increased as the gap depth decreased. To quantify the effect of gap depth, a one-factor analysis of variance with repeated measures was conducted to assess the impact of gap depth (∆I = 20, 25, 30, and 70 dB) on participants’ response latency values. A marginally significant effect for ∆I on response latency was noted, Greenhouse-Geisser = 21.98, F(1.97, 15.78) = 3.43, *p* = 0.059, η^2^ = 0.3. Although only marginally significant, the effect size did reveal that gap depth still accounted for a substantial amount (30%) of variance in response latency. There was no significant effect for ∆I on response amplitude, Greenhouse-Geisser = 0.35, F(1.83, 14.65) = 1.13, *p* = 0.34, η^2^ = 0.12, suggesting that gap depth did not affect the response to the successive stimulus (S2).

## 4. Discussions

In this study with typical adult listeners, a noninvasive method was used to determine whether one sound (S1) affected neural responsiveness to a succeeding sound (S2). The motivation was to approximate contexts in speech and music when two sounds occur rapidly, one after the other. Neural responsiveness was assessed passively with the auditory brainstem response (ABR), which is generated subcortically. Two response parameters (latency and amplitude) were measured as either the duration or depth of the silent gap between S1 and S2 was manipulated. It was reasoned that if the gap were either too brief or too shallow, the neural onset response to the succeeding stimulus would be reduced. This effect would be reflected in the ABR measurements as prolonged latency and diminished amplitude.

In experiment 1, we found that when decreasing gap duration, the latency of wave V became significantly longer. However, a change in response amplitude was not observed consistently. A similar dissociation between latency and amplitude measures has been noted for the ABR as the stimulus rate was varied. By increasing the click rate and decreasing the inter-stimulus interval, the two sounds are brought into closer temporal proximity, which is analogous to reducing the gap duration. Pratt and Sohmer [10] reported that wave V latency increased, but amplitude remained unchanged as a function of stimulus rate. Suzuki et al. [11] suggested that the lack of a relationship between latency and amplitude in rate contexts could be because the ABR consists of at least two components—a slow wave that is relatively insensitive to rate and a fast component that is affected by the rate.

Another possibility is that the variability in response amplitude was too large to observe a systematic effect of gap duration. Compared to latency, the amplitude of the ABR has long been known to be the less stable of the two measures due to moment-by-moment fluctuations in the level of the background noise [5]. Moreover, it could be that any potential relationship between response latency and amplitude was simply obscured by the variability of the response.

Although the physiological mechanism underlying the latency effect is unknown, it may reflect the disruption of neural synchrony and/or recovery from short-term adaptation [6,12]. Recall from Figure 1 that the discharge rate of a given neuron abruptly increased from its spontaneous rate at the onset of the first sound (S1). The driven rate then rapidly declined exponentially, presumably due to neural adaptation or refractoriness, over the next few milliseconds before achieving a steady-state rate [13]. The imposition of a brief silent gap enabled the neuron to recover from adaptation, thus re-arming itself in preparation for the arrival of a subsequent sound. The duration of the pause determines the extent of the recovery. In the context of the ABR, many individual neurons respond to a given stimulus. As each cell has its unique physiological properties, it seems reasonable to expect that the individual neurons recover from adaption at slightly different rates. Moreover, a brief gap would require a more extended recovery period, and that would be manifested in the ABR as prolonged latency.

The same general outcome was noted in Experiment 2, namely that wave V latency but not amplitude changed as the salience of the gap varied. In this case, the definition of the gap was modified systematically by filling it with Gaussian noise. As the gap became less prominent, the latency of the response increased. Although the effect size for gap depth (Experiment 2) was somewhat smaller than the effect size for gap duration (Experiment 1), a common mechanism was possibly responsible for both outcomes. As the gap was filled in, the neurons had progressively less opportunity to regain their synchrony or recover from adaptation, and consequently, response latency increased.

In conclusion, neural encoding is affected by the parameters of a silent gap between two successive sounds. Specifically, the latency of wave V of the ABR increased as the duration or depth of the gap decreased. The ABR originates from subcortical structures, and so this recording method may be potentially useful for monitoring the early stages of temporal processing, which may be essential for speech and language development and musical perception. The basic parameters of the ABR were influenced by the acoustic manipulations of temporal gaps consistent with results from studies where gap detection thresholds are measured behaviorally [4]. Moreover, it seems plausible to suggest that we have identified an objective index of temporal resolution worthy of exploration in clinical populations.

## Data Availability

The data presented in this study are available on request from the corresponding author. The data are not publicly available due to HIPAA and IRB constraints.
